# Derivation of a Triple Mosaic Adenovirus for Cancer Gene Therapy

**DOI:** 10.1371/journal.pone.0008526

**Published:** 2009-12-31

**Authors:** Yizhe Tang, Hongju Wu, Hideyo Ugai, Qiana L. Matthews, David T. Curiel

**Affiliations:** 1 Division of Human Gene Therapy, Departments of Medicine, Pathology, Surgery, and Obstetrics and Gynecology, and the Gene Therapy Center, University of Alabama at Birmingham, Birmingham, Alabama, United States of America; 2 Vision Science Graduate Program, University of Alabama at Birmingham, Birmingham, Alabama, United States of America; 3 Center for AIDS Research, University of Alabama at Birmingham, Birmingham, Alabama, United States of America; The University of Chicago, United States of America

## Abstract

A safe and efficacious cancer medicine is necessary due to the increasing population of cancer patients whose particular diseases cannot be cured by the currently available treatment. Adenoviral (Ad) vectors represent a promising therapeutic medicine for human cancer therapy. However, several improvements are needed in order for Ad vectors to be effective cancer therapeutics, which include, but are not limited to, improvement of cellular uptake, enhanced cancer cell killing activity, and the capability of vector visualization and tracking once injected into the patients. To this end, we attempted to develop an Ad as a multifunctional platform incorporating targeting, imaging, and therapeutic motifs. In this study, we explored the utility of this proposed platform by generating an Ad vector containing the poly-lysine (pK), the herpes simplex virus type 1 (HSV-1) thymidine kinase (TK), and the monomeric red fluorescent protein (mRFP1) as targeting, tumor cell killing, and imaging motifs, respectively. Our study herein demonstrates the generation of the triple mosaic Ad vector with pK, HSV-1 TK, and mRFP1 at the carboxyl termini of Ad minor capsid protein IX (pIX). In addition, the functionalities of pK, HSV-1 TK, and mRFP1 proteins on the Ad vector were retained as confirmed by corresponding functional assays, indicating the potential multifunctional application of this new Ad vector for cancer gene therapy. The validation of the triple mosaic Ad vectors also argues for the ability of pIX modification as a base for the development of multifunctional Ad vectors.

## Introduction

Cancer remains the second leading cause of death worldwide, and accounted for approximately 7.9 million deaths (13% of all deaths) in 2007 according to the World Health Organization (WHO, “The top 10 causes of death”). Gene therapy represents a new paradigm in the treatment of human diseases by the insertion of genetic materials into individuals' cells for therapeutic purposes [1]. Among all treatment methods for cancer, gene therapy represents a novel molecular measure that has the potential of anti-tumor efficacy with selectivity and safety [2].

To date, 65.2% of all gene therapy clinical trials have targeted cancer [3]. Among a number of cancer gene therapy strategies, however, very few of them have shown a potent therapeutic effect in human patients. In addition to the complexity and ambiguity of tumorigenesis, other obstacles that may account for the failure of many cancer gene therapy applications include the low transduction efficiency of the anti-cancer agents and the inability to transduce the entire tumor cell population; the poor selectivity and lack of long-term duration of anti-cancer agents in tumors resulting in poor therapeutic efficiency as well as safety issues. The concept of tumor cell targeting addresses these obstacles and may represent a major improvement in the development of gene therapy as an anti-cancer therapeutic. Given efficient targeting, this anti-cancer agent may not only achieve highly selective and specific tumor cell killings, but also avoid uptake by normal cells and subsequent toxicity. An *in vivo* imaging modality, which provides a means to study the distribution (e.g. accumulation, spread, retention, and etc.) of anti-cancer therapeutics, can address key issues that are fundamental to the design and test of novel anti-cancer therapies, especially for replicative virus-based therapeutics [4]–[7]. To combine these functionalities, and in an effort to create more potent and reliable anti-cancer therapeutics, we attempted to generate a multifunctional adenoviral (Ad) vector for the detection and treatment of cancer. This Ad incorporates targeting, imaging and cancer therapeutic modalities.

Ad is one of the most commonly used viral vectors in numerous cancer gene therapy clinical trials [3]. In these studies, the use of first- or second-generation adenoviral vectors has demonstrated therapeutic transgene expression, but the overall clinical efficacy has been poor due to the abovementioned limitations. To overcome the limitations, many efforts have been focused on modifying the Ad capsid on an individual cancer type basis. For example, modification of the Ad capsid with targeting ligands directed against tumor antigens boosted Ad transduction in tumor cells *in vitro* and *in vivo* [8], [9]. Other efforts aiming at Ad visualization and tracking resulted in labeling the Ad capsid with bioluminescence or fluorescence. This strategy allowed for dynamic and direct monitoring of the physical location and replication of viral particles *in vivo* [4], [10], [11]. Moreover, the incorporation of herpes simplex virus type 1 (HSV-1) thymidine kinase (TK) on the Ad capsid surface allowed for the direct functional application of this protein in suicide gene therapy and microPET imaging [12].

Our lab and others have validated the accommodating role of Ad type 5 minor capsid protein IX (pIX) for the incorporation and functional display of heterologous polypeptides and proteins, such as poly-lysine (pK) [13], green and red fluorescence proteins (GFP and RFP) [4], [10], [11], or HSV-1 TK [12], [14]. We have further demonstrated the possibility of creating a triple mosaic Ad by presenting three different heterologous epitopes at pIX locales on a single Ad vector using genetic or non-genetic modifications [15]. In this study, we explored pIX to display three functional epitopes – pK (for targeting), monomeric RFP (mRFP1) (for imaging), and HSV-1 TK (for therapeutic) on a single Ad vector in our effort of generating multifunctional Ad vectors.

## Materials and Methods

### Antibodies

The pIX-specific antibody was a kind gift from Dr. I. Dmitriev (Gene Therapy Center, University of Alabama at Birmingham). The rabbit polyclonal anti-TK antibody was a kind gift from Dr. J.M. Mathis (Gene Therapy Program, Department of Cellular Biology and Anatomy, LSU Health Sciences). The mouse anti-His_6_ monoclonal antibody was purchased from Qiagen (Valencia, CA.). The goat polyclonal anti-His_6_ antibody was purchased from Abcam (Cambridge, MA.). The anti-Flag M2 monoclonal antibody and the rabbit polyclonal anti-c-myc antibody were purchased from Sigma (St. Louis, MO.). The rabbit polyclonal anti-RFP antibody was purchased from Chemicon (Temecula, CA.). Horseradish peroxidase (HRP)-conjugated goat anti-mouse and goat anti-rabbit secondary antibodies, alkaline phosphatase (AP)-conjugated goat anti-mouse, goat anti-rabbit secondary antibodies, and electron microscopy (EM) grade 18 nm colloidal gold-conjugated donkey anti-goat antibody were purchased from Jackson ImmunoResearch Laboratories Inc. (West Grove, PA.). EM grade 10 nm gold-conjugated donkey anti-mouse, and EM grade 25 nm gold-conjugated donkey anti-rabbit secondary antibodies were purchased from Electron Microscopy Science (EMS, Ft. Washington, PA.).

### Cells

The human embryonic kidney cells (293), human lung carcinoma cells (A549), and human breast cancer cells (AU-565) were purchased from American Type Culture Collection (ATCC, Manassas, VA.). All cell lines were maintained at 37°C in a 5% CO_2_ humidified incubator. The AU-565 cells were cultured in RPMI-1640 (2 mM L-Glutamine, 10 mM HEPES, 1 mM Sodium pyruvate, 4.5 g/L glucose, 1.5 g/L sodium bicarbonate, 100 units/ml of penicillin, 100 µg/ml of streptomycin, 10% Fetal Bovine Serum). All other cell lines were cultured in Dulbecco's modified Eagle medium-Ham's F12 (50/50) medium (Sigma) containing 10% fetal calf serum (HighClone, Logan, Utah), 2 mM L-glutamine, 100 units/ml of penicillin, 100 µg/ml of streptomycin.

### Construction of Recombinant Plasmids

1. Generation of shuttle plasmids. All parental plasmids were acquired from commercial sources or have been characterized previously: pShlpIXNhe [13], pShuttle-IX-mRFP1 [4], pShuttle-IX-flag-sr39tk [12], pShuttle-CMV and pAdEasy-1 (Stratagene, La Jolla, CA). The shuttle plasmids were constructed using restriction and PCR cloning as following. pShldpIX  =  pShlpIXNhe/BglII/NheI/blunt/SL (self ligation); The PCR product (pcrIXpK) containing the coding sequence of pIX-pK fusion protein was generated by using pShlpIXNhe as a template and primers 5′-GGGGTACCGGGCGTGGTTAAGGGTG and 5′-GGGGTACCTTTATTTATGTTCTTGTCATCGTCATCCTTATAATC; pShlpIXflagpK  =  pShldpIX/KpnI + pcrIXpK/KpnI. The PCR product (pcrH6TK) containing the coding sequence of H6-sr39tk protein was generated by using pShuttle-IX-flag-sr39tk as a template and primers 5′-CTAGCTAGCCACCATCACCATCACCAT CTAGCCGGATCCGGTTC and 5′-CTAGCTAGCTCAATTAGCCTCCCCCATC; pShlpIXH6TK  =  pShlpIXNhe/NheI + pcrH6TK/NheI. The PCR product (pcrMycmRFP1) containing the coding sequence of c-myc-mRFP1 protein was generated by using pShuttle-IX-mRFP1 as a template and primers 5′- CTAGCTAGCGGCGG AGGGAGCGAGCAGAAACTCATCTCTGAAGAAGATCTGGGAAGCGCCTCCTCCGAGGACGTCAT and 5′- CTAGCTAGCTTAGGCGCCGGTGGAGTG; pShlpIXmycmRFP1  =  pShlpIXNhe/NheI + pcrMycmRFP1/NheI. All clones were verified by restriction digestion and sequencing.
2. Generation of pIX-modified adenoviral genomes by homologous recombination in *Escherichia coli* [16]. The shuttle vectors pShlpIXflagpK, pShlpIXH6TK, and pShlpIXmycmRFP1 were linearized with PmeI restriction enzyme and homologously recombined with pAdEasy-1 in electrocompetent BJ5183-AD1 (Stratagene, La Jolla, CA). The generated adenoviral genome contains IX-pK, IX-sr39tk, or IX-mRFP1 modified pIX gene in deleted E1 region. The constructs of resultant Ad plasmids pAdpIXflagpK, pAdpIXH6TK, and pAdpIXmycmRFP1 were confirmed by restriction digestion and sequencing.

### Virus Rescue, Propagation and Purification

pAdpIXflagpK, pAdpIXH6TK, and pAdpIXmycmRFP1 plasmids were linearized with PacI restriction enzyme and transfected into 293 cells grown in 25-cm^2^ flasks using Lipofectamine™ 2000 (Invitrogen, Carlsbad, CA). The cells were collected when evident cytopathic effect (CPE) was observed followed by disruption using four freeze and thaw cycles. The lysates were centrifuged at 3,000×g for 5 min at 4°C to remove cellular debris. The released viruses in the supernatant were subsequently used for further propagation until a sufficient amount of 293 cells were infected (ten 175-cm^2^ flasks). The Ad in infected cells were purified essentially as described previously [15]. In brief, the cells were lysed by four freeze and thaw cycles and centrifuged at 3,800×g for 30 min at 4°C to remove cellular debris. The cell extracts containing the viruses were loaded on the top of a 1.33/1.45 CsCl step gradient and centrifuged at 55,000×g for 3 hours at 4°C. The lower band, containing infectious virus particles, was re-centrifuged on another 1.33/1.45 CsCl step gradient at 100,000×g overnight at 4°C. The resulting band of adenoviruses was collected and dialyzed four times against 500 ml phosphate buffered saline (PBS) containing 10% glycerol, 2 hours each time. The generated Ads were designated as Ad-IX-flag-pK, Ad-IX-H6-sr39tk, and Ad-IX-myc-mRFP1. Viral particle (VP) titers were determined by spectrophotometry at OD260 using standard procedures [17].

### Generation of Triple pIX-Modified Ad by Co-Infection

Ten 175-cm^2^ flasks of 293 cells were infected with Ad-IX-flag-pK, Ad-IX-H6-sr39tk, and Ad-IX-myc-mRFP1 at a total MOI of 200–300 VP/cell. The infected cells were collected for Ad purification as described in above.

### Protein Electrophoresis and Western Blotting

5×10^9^ VP of purified viruses were boiled in Laemmli sample buffer for 5 min and separated on 4 to 15% gradient sodium dodecyl sulfate-polyacrylamide gel electrophoresis (SDS-PAGE). Separated proteins were then transferred to polyvinylidene difluoride (PVDF) membranes, which were blocked in 5% skim milk in Tris-buffered saline containing 0.05% Tween-20 (TBS-T) followed by incubation with primary antibodies (mouse anti-Flag, 1∶1000; rabbit anti-TK, 1∶500; rabbit anti-RFP, 1∶500). After washing and re-blocking, the membrane was incubated with the appropriate secondary antibodies conjugated to Horse Radish Peroxidase (HRP) at 1∶1000 dilution. The HRP signal was developed with ECL plus Western blotting detection system (GE healthcare, Little Chalfont, UK), and then detected with BioMax MR scientific imaging films (Kodak, Chalon-sur-Saone, France) and a medical film processor SRX-101A (Konica, Tokyo, Japan).

### ELISA

Solid-phase binding enzyme-linked immunosorbent assays (ELISAs) were performed essentially as described previously [18]. Briefly, 10^9^ VP of viruses were subjected to serial dilution (1, 2, 4, 8, 16, 32, 64, 128) in 100 µl of 100 mM carbonate buffer (pH 9.5), and immobilized in triplicate in a 96-well plate (Nunc Maxisorp) by overnight incubation at 4°C. After 4 washes with TBS-T and blocking with TBS-T containing 2% bovine serum albumin (BSA), the viruses were probed with primary antibody, and then AP-conjugated secondary antibodies in TBS-T containing 0.5% BSA at room temperature for 2 hours, with extensive washes and blocking in between. *p*-nitrophenyl phosphate (Sigma) was used for color development as described by the manufacturer, and light absorbance (405 nm) was obtained by a microplate reader (PowerWave HT 340, BioTek, Winooski, VT.) after incubation for 150 min at room temperature.

### Immunoelectron Microscopy

Immunoelectron microscopy was performed essentially as described previously [15]. Briefly, the viruses were adhered to 400-mesh nickel grids supported with carbon-coated Formvar film (EMS). After washing with 1% BSA/PBS twice for 10 min each, grids were probed with 1% BSA/PBS diluted primary antibodies (1∶200 M2 anti-Flag, goat anti-His_6_, and rabbit anti-c-myc) and incubated at room temperature for 1 hour. After 2 cycles of 1% BSA/PBS washes, grids were incubated with 1∶10 diluted secondary antibodies (10 nm gold-donkey anti-mouse, 18 nm gold-donkey anti-goat, and 25 nm gold-donkey anti-rabbit) at room temperature for 45 min. After fixing with 1% glutaraldehyde/PBS for 20 min, grids were subjected to negative staining in 2% uranyl acetate for 12 seconds and examined under transmission electron microscope at 60 KV in the UAB High Resolution Imaging Facility.

### Cell Binding Inhibition Assay

The binding inhibition assay was performed essentially as described previously [13]. AU-565 cells were released from flasks using Versene, washed once with PBS, and pelleted and resuspended to 0.5×10^7^ cells/ml in binding buffer (DMEM/F12 with 1% BSA). 100 µl cell aliquots were transferred into each 5 ml test tube, and were added with 50 µl of an inhibitor (soluble CAR (sCAR), heparin, or PBS). After 30 min shaking at 4°C, viruses at a multiplicity of infection (MOI) of 500 VP/cell in 50 µl binding buffer were added into each tube, and they were shaken at 4°C for 1.5 hours. The cells were washed once with 4 ml of binding buffer, and their total DNA was extracted and subjected to quantitative Real-time PCR (TaqMan) to measure Ad5 E4 copy number. Total DNA in each sample was quantified by OD260.

### Cell Killing Assay

The cell killing assay was performed essentially as described previously [12]. The lung carcinoma A549 cells were seeded on a 96-well plate at a density of 10,000 cells/well one day before Ad infection. Ad vectors carrying pIX-TK fusion proteins at various MOIs were then used to infect A549 cells. After 2 hours or 12 hours of incubation, the prodrug, ganciclovir (GCV) (GYTOVENE-IV, Roche Laboratories, Nutley, NJ), was added to the cells at a final concentration of 1 mM. After 24 or 48 hours of incubation, the viability of A549 cells was evaluated using CellTiter 96® AQ_ueous_ Non-Radioactive Cell Proliferation Assay kit (Promega, Madison, WI) and a microplate reader (PowerWave HT 340, BioTek, Winooski, VT) in accordance with the manufactories' instruction.

### Statistical Analysis

Statistical analysis was performed using two-tailed unpaired Student's *t*-tests between groups. *P* values <0.05 were considered statistically significant.

## Results

### Generation of a Triple pIX-Modified Ad by Co-Infection

**Figure 1:**
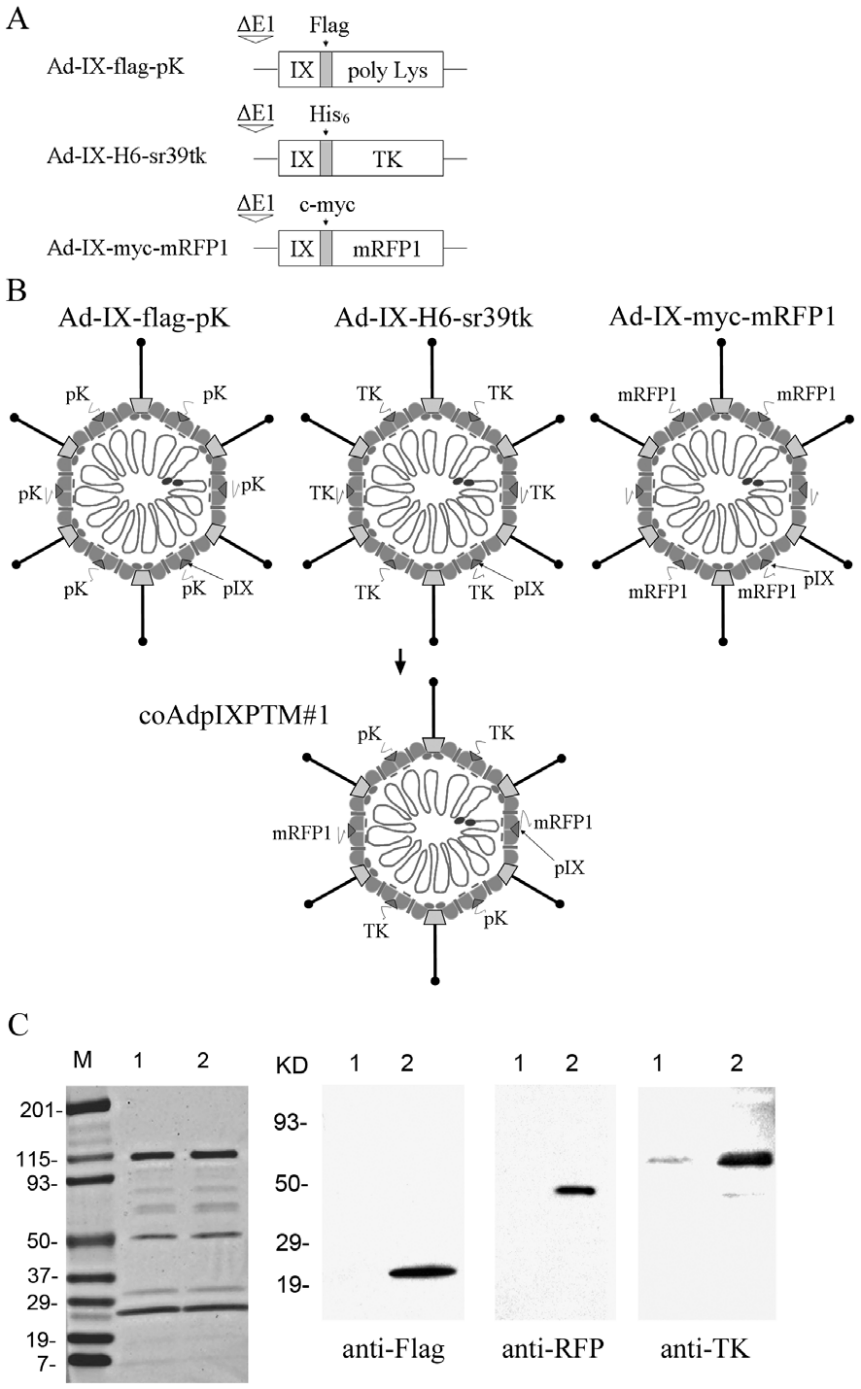
Schema of Ad pIX modifications. (A) Constructs of the modified pIX genes in genomes of three parental viruses: Ad-IX-Flag-pK, Ad-IX-H6-TK, and Ad-IX-myc-mRFP1. Modified pIX genes (tagged with the Flag, His_6_, and c-myc, respectively) were driven by the native pIX promoter. (B) Structural diagram of triple pIX-modified Ad (coAdpIXPTM#1) generated by co-infection strategy. pK, TK, and mRFP1 peptides/proteins were incorporated in the C termini of pIX. (C) Western blotting analysis of Ad vector containing triple pIX modifications. 5×10^9^ VPs of CsCl-purified control Ad5 (lane 1) and the triple pIX-modified coAdpIXPTM#1 (lane 2) were subjected to SDS-PAGE. The separated proteins were stained with Gelcode Blue (Pierce, Rockford, IL.) to detect the total viral proteins (left panel) or probed with anti-Flag, anti-RFP, or anti-TK antibody (right panel).

In order to generate the triple pIX-modified Ad carrying poly-lysine (pK), HSV-1 thymidine kinase mutant (HSV-1 sr39tk), and monomeric red fluorescent protein (mRFP1) by co-infection, three parental viruses were generated first: Ad-IX-Flag-pK, Ad-IX-H6-TK, and Ad-IX-myc-mRFP1. These three viruses express pIX-pK, pIX-TK, or pIX-mRFP1 fusion protein with Flag, His_6_, or c-myc tag, respectively (Fig. 1A). Unless specified, all viruses used in the study are replication-incompetent (E1/E3 deleted), and the transcription of modified pIX genes in each parental virus was driven by the endogenous pIX promoter. In addition, pIX-modified Ads only express the pIX fusion protein since the native pIX genes have been replaced with the modified pIX genes. The three parental viruses were used to co-infect 293 cells that support Ad replication to generate the triple pIX-modified virus. Since the three different pIX fusion proteins were all expressed and could be assembled into each viral particle, the resultant virions were expected to contain a mixture of three types of pIX fusion proteins (Fig. 1B). The generated and CsCl-purified Ad progenies (designated as coAdpIXPTM#1) had a normal yield compared to single pIX-modified Ads in our laboratory (data not shown). The incorporation of pIX fusion proteins was analyzed by Western blotting with anti-Flag, anti-RFP, and anti-TK antibodies. The detection of pIX fusion protein bands with expected molecular masses confirmed that three pIX fusion proteins (i.e. pIX-pK, pIX-TK, and pIX-mRFP1) were contained in the viral particles of purified viral pool (Fig. 1C).

### Surface Display of Modified pIXs in Ad Particles

**Figure 2:**
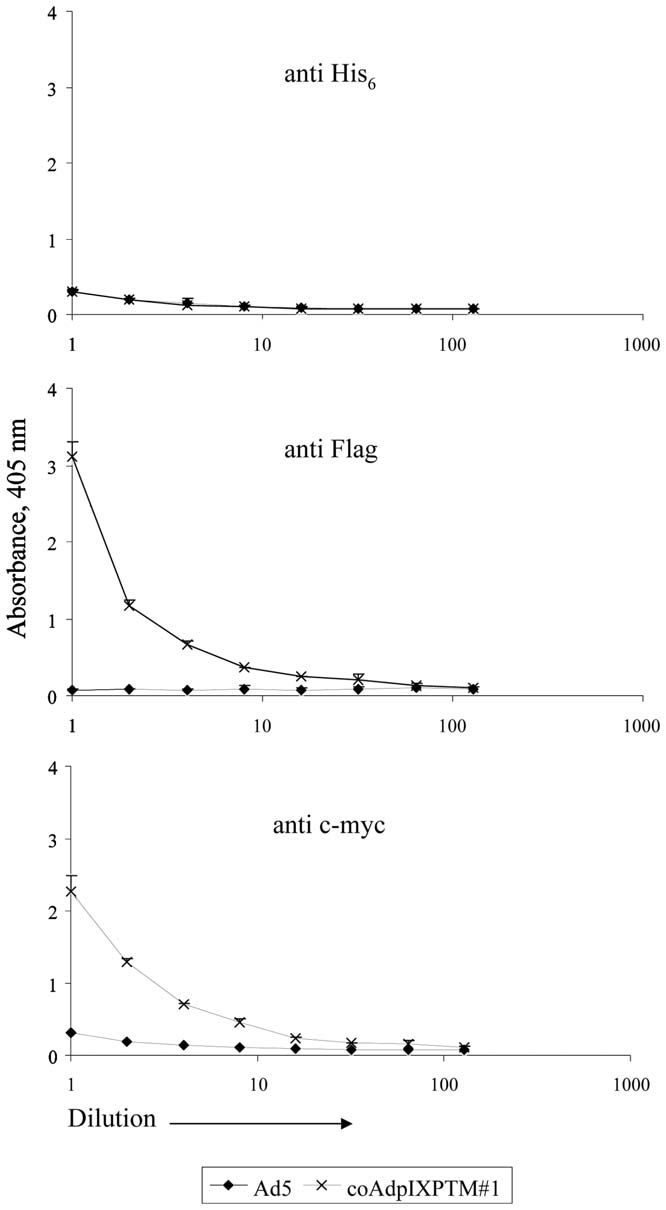
Incorporation and presentation of modified IX proteins on the Ad capsid. ELISA analysis of Ad vector containing triple pIX modifications. 10^9^ VPs of CsCl-purified Ad5 (negative control) or coAdpIXPTM#1 were subjected to a serial dilution (1, 1/2, 1/4, 1/8, 1/16, 1/32, 1/64, 1/128) and immobilized on an ELISA plate. The viruses were probed with anti-Flag (1∶2000), anti-His_6_ (1∶1000), and anti-c-myc antibodies (1∶1000), followed by incubation with alkaline phosphatase-conjugated secondary antibodies (1∶1000). After color development, the light absorbance was measured and plotted on the Y-axis against the viral concentrations. Each point represents the mean and standard deviation (SD) of triplicate determinations. Some error bars standing for SD are smaller than their symbols.

To test whether or not the polypeptides/proteins incorporated into the C-terminus of pIX were presented on the viral surface and accessible to antibodies, we performed enzyme-linked immunosorbent assays (ELISAs). In the ELISA experiment, anti-Flag, anti-His_6_, and anti-c-myc antibodies were used to detect the three types of modified pIXs. The control Ad5 containing wild type pIX was not recognized by any of these antibodies. The coAdpIXPTM#1 viruses were recognized by anti-Flag, and anti-c-myc antibodies (Fig. 2). However, the viruses could not be recognized by either anti-His_6_ antibody (Fig. 2) or anti-TK antibody (data not shown). This result suggests that the pK and mRFP1 were incorporated into the triple pIX-modified Ad vectors. The insignificant binding of anti-His_6_ or anti-TK antibody to the viruses indicates that TK was either not efficiently surface exposed on the viral capsids or the accessibility of these two antibodies to TK proteins in such a configuration was insufficient to be detected in ELISA.

### Mosaicism of the Triple pIX-Modified Ad

**Figure 3:**
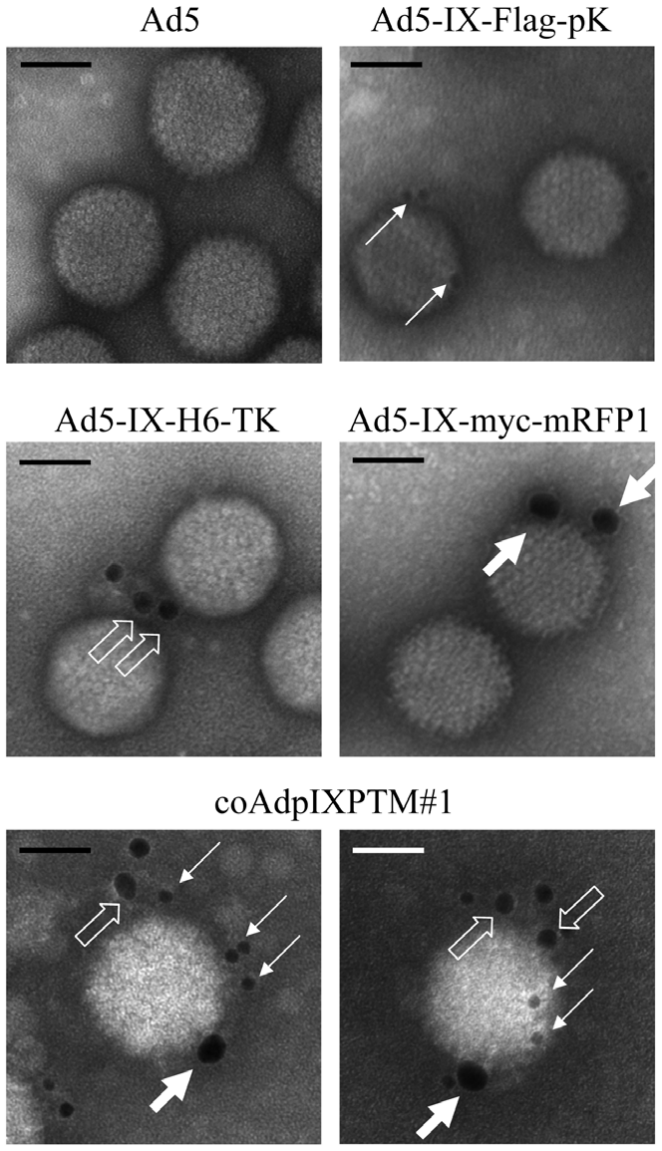
Immuno-gold electron microscopy on pIX-modified Ad vectors. Control or pIX-modified Ad vectors were loaded onto EM grids, probed with gold nanoparticle conjugated antibodies, and observed under electron microscope at 60 KV. Mouse anti-Flag, goat anti-His_6_, and rabbit anti-c-myc primary antibodies were used for detecting three kinds of tags on pK, HSV-1 TK, and mRFP1, respectively. 10 nm gold-donkey anti-mouse, 18 nm gold-donkey anti-goat, and 25 nm gold-donkey anti-rabbit secondary antibodies were used for subsequent gold labeling of Ad particles. Solid thin arrows indicate 10 nm gold particles, empty arrows indicate 18 nm gold particles, and solid thick arrows indicate 25 nm gold particles. The scale bar in each panel represents 50 nm in length.

Western blotting and ELISA analysis of the purified viral particles demonstrated that three types of pIX fusion proteins were incorporated into the Ad particles produced by co-infection (Fig. 1). To examine whether a single Ad viral particle could display all three types of functional proteins, we performed immunogold electron microscopy to directly visualize pIX-modified Ad particles. Anti-Flag, anti-His_6_ and anti-c-myc antibodies were used to detect pIX-pK, pIX-TK and pIX-mRFP1 proteins in the viral particles, respectively, followed by three corresponding secondary antibodies conjugated with 10, 18, and 25 nm gold particles, respectively. As shown in Figure 3, no gold particle was bound to the control Ad5 displaying wild type pIX, while the Ad viral particles containing a single pIX fusion protein (Ad-IX-Flag-pK, Ad-IX-H6-TK, and Ad-IX-myc-mRFP1) were stained by corresponding gold particles. The triple modified coAdpIXPTM#1 viruses were recognized by anti-Flag, anti-His_6_ and anti-c-myc antibodies and labeled with 10, 18 and 25 nm gold particles. The results demonstrated that three types of pIX fusion proteins could coexist on one single viral particle and were exposed on the viral surface.

It is noteworthy that, although there are 240 copies of pIX molecules in each viral particle, only a few gold particle-conjugated antibodies were bound on the pIX-modified Ad viral particles. This data was consistent with previous reports [15], [19], [20]. This is probably due to the relative low accessibility of pIX from capsid surface [20]–[22] and spatial hindrance effect of antibody-conjugated gold particles against each other during staining. We chose large gold particles for easier differentiation among three epitopes, which have even a higher antibody/gold ratio and a higher spatial hindrance effect than that of small gold particles which are commonly used. In addition, anti-His_6_ antibody does not efficiently bind pIX-TK as shown in Figure 2. Furthermore, the low efficiency of triple staining may be another reason of the scattered staining pattern shown in Figure 3. Therefore, it is not surprising that the frequency of triply-labeled Ads is low - only about 1% viral particles were stained with all three types of gold particles (Table 1).

**Table 1:** Summary of immunogold staining of coAdpIXPTM#1.

Epitopes to be detected	Flag	His_6_	c-myc	Flag+His_6_	Flag+myc	His_6_+myc	Triple
Properties of gold-labeled antibodies*							
Size of gold particle (nm)	10	18	25				
Antibody/gold ratio	7–12	N/A	115–180				
Statistics of staining							
The number of viral particles with positive staining	210	13	12	12	11	2	2
Percentage of viral particles with positive staining over total viral particles (%)	98.13	6.07	5.61	5.61	5.14	0.93	0.93

N/A  =  not available.

*Provided by the manufacturer.

The counting was performed in eleven electron microscopy images that totally contain 214 viral particles.

### Targeting Activity of Incorporated Poly-Lysine on the Triple pIX Mosaic Ad

Ad5 has been demonstrated to bind its primary cellular receptor, the coxsackie B virus and adenovirus receptor (CAR), as the initial step of infection via its fiber knob protein [23]. The poly-lysine (pK) incorporated into pIX locales in AdLucIXpK has been shown to mediate Ad interaction with cellular heparan sulfate proteoglycans (HSPGs), resulting in CAR-independent virus-cell binding and transduction [13]. Therefore, in order to examine whether the pK peptides incorporated into the triple pIX mosaic Ad vector had similar targeting activity to HSPGs, we performed cell binding and inhibition assays with CAR-deficient (low level of CAR) AU-565 cells in the presence or absence of heparin or soluble CAR (sCAR) protein.

**Figure 4:**
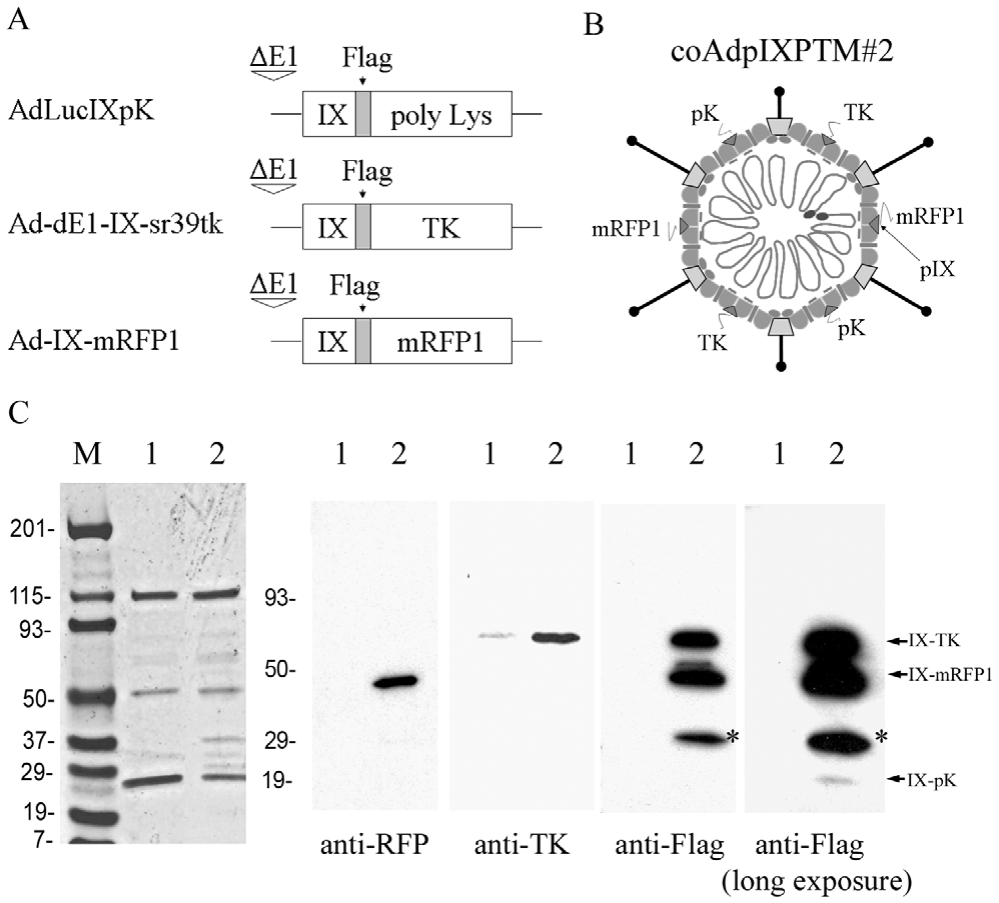
Western blotting analysis of coAdpIXPTM#2. (A) Constructs of the modified pIX genes in genomes of three parental viruses: AdLucIXpK, Ad-dE1-IX-sr39tk, and Ad-IX-mRFP1. Modified pIX genes (tagged with the Flag) were driven by the native pIX promoter. (B) Structural diagram of triple pIX-modified Ad (coAdpIXPTM#2) generated by co-infection strategy. (C) 5×10^9^ VPs of CsCl-purified control Ad5 (lane 1) and the triple pIX-modified coAdpIXPTM#2 (lane 2) were subjected to SDS-PAGE. The separated proteins were stained with Gelcode Blue (on the left) or probed with anti-Flag, anti-RFP, or anti-TK antibody. A long-exposure of the anti-Flag blot was included to show pIX-pK protein, which is incorporated into Ad particles at a very low level. The “*” asterisks indicate degradation products of pIX-mRFP1 fusion protein.

Another version of the triple pIX-modified Ad vector was created using three previously characterized parental pIX-modified viruses: AdLucIXpK [13], Ad-dE1-IX-sr39tk [12], and Ad-IX-mRFP1 [4] (Flag tags were present in all three pIX fusion proteins). The incorporation of pIX-pK, pIX-TK, and pIX-mRFP1 proteins on the derived triple mosaic Ad (designated as coAdpIXPTM#2) was verified by Western blotting on the purified viruses using anti-Flag, anti-RFP, and anti-TK antibodies (Fig. 4C).

**Figure 5:**
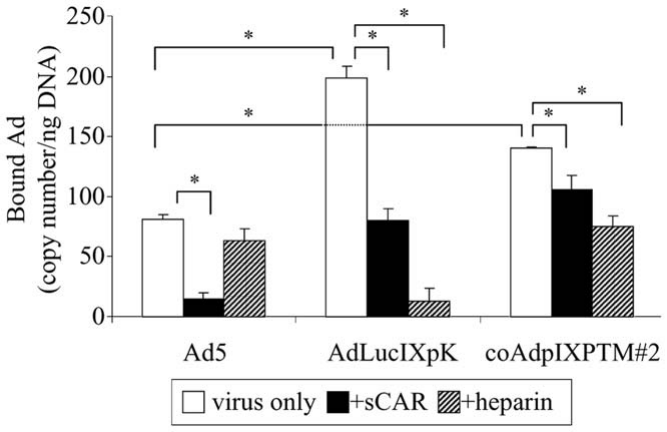
Cell Binding of the triple pIX-modified Ad vector. AU-565 cells were incubated with Ad5, AdLucIXpK, or coAdpIXPTM#2 at an MOI of 500 VP/cell in the presence of 500 µg/ml heparin or 200 µg/ml sCAR protein at 4°C. Bound Ad particles were quantified by quantitative real-time PCR and normalized with total cellular DNA. Each bar represents the mean and SD of triplicate determinations, and asterisks indicate significant difference between specified groups.

It was observed that the positive control AdLucIXpK, in which all pIX molecules were modified by pK, exhibited as much as two times of cell binding on CAR-deficient AU-565 cells compared to wild type Ad5. The triple mosaic Ad5 (coAdpIXPTM#2), in which only a portion of pIX molecules contained pK modification, showed approximately 1.5-time higher cell binding activity compared to wild type Ad5 (Fig. 5). In addition, more than 90% cell binding ability of AdLucIXpK and 50% of the coAdpIXPTM#2 were inhibited by free heparin at a final concentration of 500 ug/ml, while the cell binding of the control Ad5 was not significantly affected (Fig. 5). The addition of 200 µg/ml sCAR inhibited more than 80% cell binding of control Ad5, while having a relatively modest effect on AdLucIXpK (60%) and coAdpIXPTM#2 (25%) (Fig. 5). Together, our data suggest that the pK peptides displayed on coAdpIXPTM#2 capsids could mediate CAR-independent cell targeting through the interaction with cellular heparan sulfate receptors.

### Enzymatic Activity of Incorporated TK on Triple pIX Mosaic Ad

**Figure 6:**
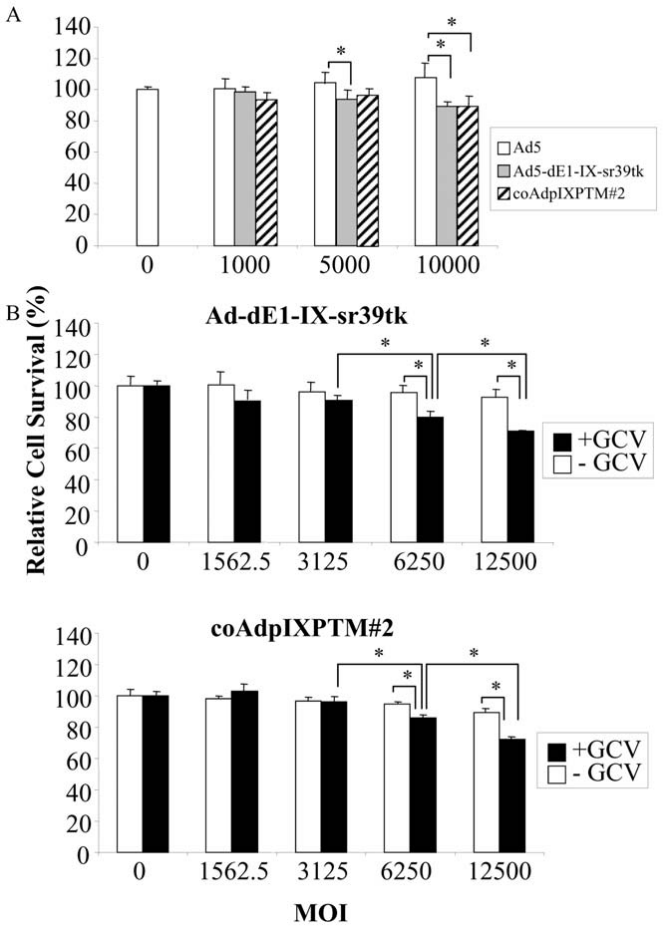
Cell killing effect of HSV-1 TK incorporated Ad vectors. (A) A549 cells were infected with Ad5 (negative control), Ad-dE1-IX-sr39tk, or coAdpIXPTM#2 at various MOIs. 2 hours later, prodrug GCV was added onto cells with the final concentration at 1 mM. 24 hours later, the cell killing activity induced by converted GCV mediated through pIX-TK was evaluated by MTS assay. Cells infected with viruses were normalized with that of uninfected cells as relative cell survival. (B) A549 cells were infected with single pIX-modified Ad-dE1-IX-sr39tk or coAdpIXPTM#2 at various MOIs. 12 hours later, GCV was added onto cells at the final concentration of 1 mM. 48 hours later, the cell killing activity of TK/GCV was evaluated as same as described above. Asterisks indicate a significant difference between specified groups.

HSV-1 TK protein can be genetically incorporated into Ad at pIX locales with its native enzymatic activity retained, which can be used for cancer gene therapy with prodrug ganciclovir (GCV), and can be utilized for *in vitro* and *in vivo* imaging when coupled with microPET system [12], [14]. To investigate whether the pIX-TK fusion proteins could function normally, we evaluated TK-induced cytotoxicity in the presence of GCV at a pharmacologic concentration by MTS assay. To evaluate the pIX-TK fusion protein directly released from Ad particles after successful cellular entry, lung carcinoma A549 cells were used, in which the replication of non-replicative Ad and the pIX gene expression are minimal. A549 cells were infected with single pIX-modified Ad (Ad-dE1-IX-sr39tk) or triple pIX mosaic Ad (coAdpIXPTM#2) at various MOIs. GCV was added in infected cells two hours post infection, and the cell death mediated by pIX-TK fusion protein was evaluated by MTS assay 24 hours thereafter. The data suggested that there was significant GCV-associated cytotoxicity induced by either Ad-dE1-IX-sr39tk or coAdpIXPTM#2 infection at a high MOI (10,000 VP/cell) (Fig. 6A). However, at a lower MOI (5000 VPs/cell), only Ad-dE1-IX-sr39tk infection showed GCV-induced significant cytotoxicity. This indicated that the pIX-TK activity in the triple pIX-modified Ad was lower than that of single pIX-TK-modified Ad, which may be attributable to the copy number differences of pIX-TK incorporated in the viral particles.

We then extended the viral incubation time (12 hours) prior to GCV addition as well as the GCV treatment time (48 hours). We found that both Ad-dE1-IX-sr39tk and coAdpIXPTM#2 induced significant cytotoxicity at a medium and a high MOI, indicating that a longer time of viral incubation and GCV treatment could enhance the pIX-TK mediated cytotoxicity (Fig. 6B). Altogether, our data suggested that there was a modest effect of the pIX-TK in the triple pIX-modified Ad since high MOI had to be used. The data also suggested that the triple-pIX modified Ad was functional in cell killing.

### Fluorescent Activity of Incorporated mRFP1 on the Triple pIX Mosaic Ad

**Figure 7:**
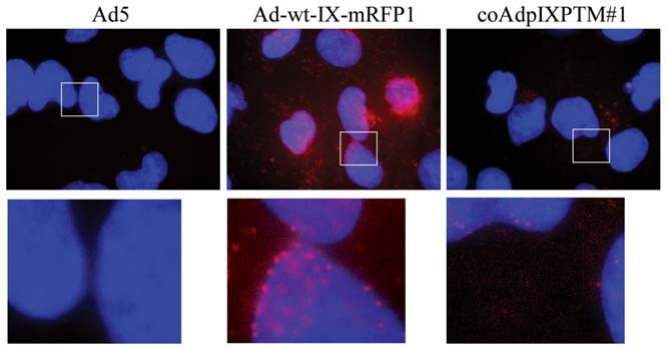
Fluorescence microscopy of cells infected with the triple pIX mosaic Ad vector. A549 cells were infected with wild type Ad5, Ad-wt-IX-mRFP1 (containg wild type E1 promoter), or coAdpIXPTM#1 at an MOI of 10,000 VP/cell, and the fluorescent signal of pIX-mRFP1 was monitored 1 hour post infection (nuclei were counterstained with Hoechst 33342). Fluorescent images were captured using an Olympus IX-70 microscope with a 100X objective (oil lens). The enlarged image of each rectangle area was presented underneath.

Ad vectors labeled with fluorescent proteins (e.g. GFP and mRFP1) have been demonstrated to be useful imaging tools to monitor and evaluate the viral replication and localization both *in vitro* and *in vivo* [4], [10], [11], [24]. To confirm the fluorescent signal of pIX-mRFP1 proteins displayed on the triple pIX mosaic Ad, A549 cells were infected with the virus, and the mRFP1 activity was monitored by fluorescence microscopy one hour post infection. The result showed that pIX-mRFP1 proteins associated with the triple pIX mosaic Ad capsid (coAdpIXPTM#1) retained their fluorescent activity, but was lower than that from Ad-wt-IX-mRFP1 (Fig. 7).

## Discussion

Treatment of tumors remains a lofty task and a long time battle in our medical community. With the hope of overcoming the inefficiency and toxicity of conventional therapies, cancer gene therapy has provided diverse strategies and molecular techniques as armors in the battle. In our previous work we have utilized approaches based upon dual expression cassettes to achieve fiber mosaic particles [25]–[27]. In these studies a frequent technical challenge has been the achievement of an expression stoichiometry that allowed mosaic particles with full representation of the capsid protein variants. In our current studies, we have advanced this aspect of the approach to better optimize adequate expression of each capsid protein construction.

Specifically, in this study, we have adopted a co-infection strategy to derive mosaic Ad vectors that embody the multifunctional principle central to our goal. The derived Ad vector carried three distinct types of heterologous peptides/proteins at the minor capsid protein IX locales, which are pK, HSV-1 TK, and mRFP1, whereby function as targeting, tumor cell killing, and imaging motifs and represent key features of an anti-cancer therapeutics. The incorporation of three types of pIX fusion protein in the triple mosaic Ad vector was demonstrated by Western blot and ELISA. Electron microscopy was used to demonstrate that these three types of pIX fusion protein were exposed on the viral surface and could coexist in a single viral particle. Due to the nature of aggregation of pIX-modified proteins [28], any affinity purification-based method would be weak in the demonstration of the mosaicism of three types of pIX on Ad capsid. Therefore, electron microscopy is the most straightforward and unambiguous method in the interpretation of the mosaicism despite low staining efficiency. In addition, the pIX-pK displayed on the Ad capsid could mediate HSPGs dependent binding to human breast cancer cells (AU-565). Due to the lack of a proper reporter gene in the triple pIX-modified Ad, we could not perform gene transfer assays to validate the gene transfer enhancement of this virus. However, a previous publication of our group showed that the pK-induced cell binding enhancement correlates with the transduction enhancement in several CAR-deficient cell lines (including AU-565 cell line) [13]. Moreover, the viral capsid-incorporated pIX-TK could induce cytotoxicity with GCV in lung carcinoma A549 cells, and the capsid-incorporated pIX-mRFP1 could signal the localization of viral particles in virus-infected cells. The activities of three types of pIX fusion proteins were relatively low compared with their parental single pIX-modified Ad vectors. These may be attributable to several reasons. First, theoretically each type of pIX fusion protein occupies one third of the total pIX locales in the triple pIX mosaic Ad, resulting in a lower copy number of each type of pIX fusion than single pIX-modified virus. Second, pIX fusion proteins, at least pIX-EGFP and pIX-mRFP1, may be aggregated to some extent in the Ad capsid [28]. Therefore, three types of pIX fusion proteins may not be compatible with each other and are susceptible to aggregation during viral assembly, which lowers the activity of incorporated functional motifs. Taken together, these Ad capsid-incorporated heterologous peptides/proteins, i.e. pK, TK, and mRFP1, retained their functionalities and could therefore serve as targeting, cell killing, and imaging motifs, respectively. These results argued for the platform potential of the triple pIX-modified Ad vector in building a multifunctional nanoparticle in cancer gene therapy.

Furthermore, we would like to point out that we also explored a genetic method as previously described [15] to construct the triple pIX mosaic virus by replacing the wild type pIX gene with three modified pIX genes, i.e. IX-pK, IX-TK, and IX-mRFP1, in the Ad genome. Even though such generated triple pIX mosaic Ad was successfully rescued and was appropriately propagated, severe genome rearrangement occurred as analyzed by restriction enzyme digestion (data not shown). These results suggest that the insertion of three modified pIX genes with a total length of ∼5kb in the deleted E1 region compromised the genomic stability, probably due to the homologous recombination among three adjacent pIX gene sequences.

Several factors need to be considered before utilizing the triple mosaic Ad vector for cancer therapy. One factor is the stoichiometry of three different pIXs in the Ad viral particle. The three types of modified pIXs, i.e. pIX-pK, pIX-TK and pIX-mRFP1, have different properties such as size, surface charge, structure and conformation. Therefore, the modified pIXs are not likely to be equally assembled even if equal expression levels were achieved (for example, using a single set of *in cis* components to drive the co-expression of three different pIX proteins, such as the Foot-and-mouth disease virus 2A (FMDV-2A) elements [29]–[31]). As we have observed in the anti-Flag (detects all modified pIXs) staining in Figure 4, pIX-mRFP1 and pIX-TK fusion proteins were much more abundantly incorporated into the Ad particles compared with pIX-pK protein. Nevertheless, a potential merit of the co-infection strategy is that the incorporation levels of three different pIXs in the triple mosaic Ad vector could be adjusted to meet different requirements in distinct applications, by altering the MOI of each parental virus.

In addition to carrying targeting, therapeutic, and imaging motifs (pK, HSV-1 TK, and mRFP1 in this study), the flexibility of our multifunctional Ad vector platform is beneficial to the design of viral vectors to fulfill other sophisticated tasks. For example, better understanding of organ and tissue's physical barriers makes it possible to deliver viral vectors *in vivo* more efficiently than before by utilizing various transcytosis machineries [32]–[36], which need an extra relaying motif for transcytosis in addition to the targeting ligand. Other tasks include but not limited to: a dual imaging system coupled with Fluorescence Resonance Energy Transfer (FRET) technology to maximize detection reliability and minimize background [35], [36]; a double targeting anti-cancer agent to achieve enhancement in delivery and specificity [27].

Taken together, the potential of our radically designed and novel mosaic adenoviral vectors has been clearly suggested. Our study presented herein is a solid argument for the concept of building a multifunctional cancer therapeutic particle based on triple modifications on Ad, bearing in mind that much work is still in need in terms of combining and optimizing the targeting/re-targeting, imaging, and therapeutic functionalities within specific tumor scenarios.

## References

[1] Edelstein ML, Abedi MR, Wixon J, Edelstein RM (2004). Gene therapy clinical trials worldwide 1989–2004-an overview.. J Gene Med.

[2] Wadhwa PD, Zielske SP, Roth JC, Ballas CB, Bowman JE (2002). Cancer gene therapy: scientific basis.. Annu Rev Med.

[3] Edelstein ML, Abedi MR, Wixon J (2007). Gene therapy clinical trials worldwide to 2007–an update.. J Gene Med.

[4] Le LP, Le HN, Dmitriev IP, Davydova JG, Gavrikova T (2006). Dynamic monitoring of oncolytic adenovirus in vivo by genetic capsid labeling.. J Natl Cancer Inst.

[5] Yang M, Baranov E, Jiang P, Sun FX, Li XM (2000). Whole-body optical imaging of green fluorescent protein-expressing tumors and metastases.. Proc Natl Acad Sci U S A.

[6] Mathis JM, Williams BJ, Sibley DA, Carroll JL, Li J (2006). Cancer-specific targeting of an adenovirus-delivered herpes simplex virus thymidine kinase suicide gene using translational control.. J Gene Med.

[7] Mocanu JD, Yip KW, Alajez NM, Shi W, Li JH (2007). Imaging the modulation of adenoviral kinetics and biodistribution for cancer gene therapy.. Mol Ther.

[8] Glasgow JN, Everts M, Curiel DT (2006). Transductional targeting of adenovirus vectors for gene therapy.. Cancer Gene Ther.

[9] Waehler R, Russell SJ, Curiel DT (2007). Engineering targeted viral vectors for gene therapy.. Nat Rev Genet.

[10] Le LP, Everts M, Dmitriev IP, Davydova JG, Yamamoto M (2004). Fluorescently labeled adenovirus with pIX-EGFP for vector detection.. Mol Imaging.

[11] Meulenbroek RA, Sargent KL, Lunde J, Jasmin BJ, Parks RJ (2004). Use of adenovirus protein IX (pIX) to display large polypeptides on the virion–generation of fluorescent virus through the incorporation of pIX-GFP.. Mol Ther.

[12] Li J, Le L, Sibley DA, Mathis JM, Curiel DT (2005). Genetic incorporation of HSV-1 thymidine kinase into the adenovirus protein IX for functional display on the virion.. Virology.

[13] Dmitriev IP, Kashentseva EA, Curiel DT (2002). Engineering of adenovirus vectors containing heterologous peptide sequences in the C terminus of capsid protein IX.. J Virol.

[14] Matthews QL, Sibley DA, Wu H, Li J, Stoff-Khalili MA (2006). Genetic incorporation of a herpes simplex virus type 1 thymidine kinase and firefly luciferase fusion into the adenovirus protein IX for functional display on the virion.. Mol Imaging.

[15] Tang Y, Le LP, Matthews QL, Han T, Wu H (2008). Derivation of a triple mosaic adenovirus based on modification of the minor capsid protein IX.. Virology.

[16] He TC, Zhou S, da Costa LT, Yu J, Kinzler KW (1998). A simplified system for generating recombinant adenoviruses.. Proc Natl Acad Sci U S A.

[17] Maizel JV, White DO, Scharff MD (1968). The polypeptides of adenovirus. I. Evidence for multiple protein components in the virion and a comparison of types 2, 7A, and 12.. Virology.

[18] Wu H, Han T, Belousova N, Krasnykh V, Kashentseva E (2005). Identification of sites in adenovirus hexon for foreign peptide incorporation.. J Virol.

[19] Akalu A, Liebermann H, Bauer U, Granzow H, Seidel W (1999). The subgenus-specific C-terminal region of protein IX is located on the surface of the adenovirus capsid.. J Virol.

[20] Vellinga J, Rabelink MJ, Cramer SJ, van den Wollenberg DJ, Van der Meulen H (2004). Spacers increase the accessibility of peptide ligands linked to the carboxyl terminus of adenovirus minor capsid protein IX.. J Virol.

[21] Saban SD, Nepomuceno RR, Gritton LD, Nemerow GR, Stewart PL (2005). CryoEM structure at 9A resolution of an adenovirus vector targeted to hematopoietic cells.. J Mol Biol.

[22] Campos SK, Barry MA (2004). Rapid construction of capsid-modified adenoviral vectors through bacteriophage lambda Red recombination.. Hum Gene Ther.

[23] Shenk T (1996). Adenoviridae: The Viruses and Their Replication; Fields BN, Knipe DM, Howley PM, editors..

[24] Le LP, Li J, Ternovoi VV, Siegal GP, Curiel DT (2005). Fluorescently tagged canine adenovirus via modification with protein IX-enhanced green fluorescent protein.. J Gen Virol.

[25] Takayama K, Reynolds PN, Short JJ, Kawakami Y, Adachi Y (2003). A mosaic adenovirus possessing serotype Ad5 and serotype Ad3 knobs exhibits expanded tropism.. Virology.

[26] Pereboeva L, Komarova S, Mahasreshti PJ, Curiel DT (2004). Fiber-mosaic adenovirus as a novel approach to design genetically modified adenoviral vectors.. Virus Res.

[27] Tsuruta Y, Pereboeva L, Glasgow JN, Rein DT, Kawakami Y (2007). A mosaic fiber adenovirus serotype 5 vector containing reovirus sigma 1 and adenovirus serotype 3 knob fibers increases transduction in an ovarian cancer ex vivo system via a coxsackie and adenovirus receptor-independent pathway.. Clin Cancer Res.

[28] Ugai H, Wang M, Le LP, Matthews DA, Yamamoto M (2009). In Vitro Dynamic Visualization Analysis of Fluorescently Labeled Minor Capsid Protein IX and Core Protein V by Simultaneous Detection.. J Mol Biol.

[29] Donnelly ML, Gani D, Flint M, Monaghan S, Ryan MD (1997). The cleavage activities of aphthovirus and cardiovirus 2A proteins.. J Gen Virol.

[30] Donnelly ML, Luke G, Mehrotra A, Li X, Hughes LE (2001). Analysis of the aphthovirus 2A/2B polyprotein ‘cleavage’ mechanism indicates not a proteolytic reaction, but a novel translational effect: a putative ribosomal ‘skip’.. J Gen Virol.

[31] Szymczak AL, Workman CJ, Wang Y, Vignali KM, Dilioglou S (2004). Correction of multi-gene deficiency in vivo using a single ‘self-cleaving’ 2A peptide-based retroviral vector.. Nat Biotechnol.

[32] Bobardt MD, Chatterji U, Selvarajah S, Van der Schueren B, David G (2007). Cell-free human immunodeficiency virus type 1 transcytosis through primary genital epithelial cells.. J Virol.

[33] Bomsel M (1997). Transcytosis of infectious human immunodeficiency virus across a tight human epithelial cell line barrier.. Nat Med.

[34] Di Pasquale G, Chiorini JA (2006). AAV transcytosis through barrier epithelia and endothelium.. Mol Ther.

[35] Tang Y, Han T, Everts M, Zhu ZB, Gillespie GY (2007). Directing adenovirus across the blood-brain barrier via melanotransferrin (P97) transcytosis pathway in an in vitro model.. Gene Ther.

[36] Zhu ZB, Makhija SK, Lu B, Wang M, Rivera AA (2004). Transport across a polarized monolayer of Caco-2 cells by transferrin receptor-mediated adenovirus transcytosis.. Virology.

